# Understanding the condition of disease prevention and control workforce by disciplines, duties, and work stress during the COVID-19 pandemic: A case from Beijing disease prevention and control system

**DOI:** 10.3389/fpubh.2022.861712

**Published:** 2022-08-18

**Authors:** Ayan Mao, Yujie Yang, Yueli Meng, Qianhang Xia, Shuyan Jin, Wuqi Qiu

**Affiliations:** ^1^Institute of Medical Information, Chinese Academy of Medical Sciences and Peking Union Medical College, Beijing, China; ^2^Affiliated Shenzhen Maternity & Child Healthcare Hospital, Southern Medical University, Shenzhen, China

**Keywords:** disease prevention and control, COVID-19, front-line workers, discipline, work stress, Beijing

## Abstract

**Objective:**

The duties, discipline cross-complementation, and work stress of professional staff during the COVID-19 pandemic are analyzed and summarized to provide a scientific basis for workforce allocation and reserve in respect of infectious disease prevention and control in the disease prevention and control (DPC) system.

**Method:**

The cross-sectional survey was made in April-May 2021 on professional staff in the Beijing DPC system by way of typical + cluster sampling. A total of 1,086 staff were surveyed *via* electronic questionnaire, which was independently designed by the Study Group and involves three dimensions, i.e., General Information, Working Intensity & Satisfaction, and Need for Key Capacity Building. This paper focuses on the former two dimensions: General Information, Working Intensity, and Satisfaction. The information collected is stored in a database built with Microsoft Excel 2010 and analyzed statistically with SPSS 22.0. The results are expressed in absolute quantities and proportions. Assuming that the overload of work stress is brought by incremental duties and cross-discipline tasks, a binary logistic regression model is constructed.

**Results:**

Among the 1086 staff surveyed, 1032 staff were engaged in COVID-19 prevention and control works, and they can be roughly divided into two groups by their disciplines: Public Health and Preventive Medicine (hereinafter referred to P, 637 staff, as 61.72%) and Non-Public Health and Preventive Medicine (hereinafter referred to N-P, 395 staff, as 38.28%). During the COVID-19 pandemic, the 1,032 staff assumed a total of 2239 duties, that is, 2.17 per person (PP), or 2.45 PP for the P group and 1.72 PP for the N-P group. As to four categories of duties, i.e., Spot Epidemiological Investigation and Sampling, Information Management and Analysis, On-site Disposal, Prevention, Control Guidance, and Publicity, the P group accounts for 76.14, 78.50, 74.74, and 57.66%, respectively, while the N-P group accounts for 23.86, 21.50, 25.26, and 42.34%, respectively. Obviously, the former proportions are higher than the latter proportions. The situation is the opposite of the Sample Detection and Other Works, where the P group accounts for 25.00 and 31.33%, respectively, while the N-P group accounts for 75.00 and 68.67%, respectively. The analysis of work stress reveals that the P group and N-P group have similar proportions in view of full load work stress, being 48.67 and 50.13%, respectively, and the P group shows a proportion of 34.38% in view of overload work stress, apparently higher than the N-P group (24.05%). Moreover, both groups indicate their work stresses are higher than the pre-COVID-19 period levels. According to the analysis of work stress factors, the duty quantity and cross-discipline tasks are statistically positively correlated with the probability of overload work stress.

**Conclusion:**

The front-line staff in the DPC system involved in the COVID-19 prevention and control primarily fall in the category of Public Health and Preventive Medicine discipline. The P group assumes the most duties, and the N-P group serves as an important cross-complement. The study results indicate that the prevention and control of same-scale epidemic require the duty post setting at least twice than usual. As to workforce recruitment, allocation, and reserve in respect of the DPC system, two solutions are optional: less addition of P staff, or more addition of N-P staff. A balance between P and N-P staff that enables the personnel composition to accommodate both routine DPC and unexpected epidemic needs to be further discussed.

## Background

The COVID-19 pandemic has brought huge impacts to the economy, societies, and health systems worldwide (1). China has stuck to the dynamic zero-COVID approach as its line of defense against the epidemic since the outbreak, which aims to reduce the number of transmission cases in the country to zero through a set of pandemic control and prevention measures, such as quarantining close contacts, locking down residential communities with transmissions and imposing travel restrictions, etc. (2). As the core and professional workforce in the fight against the pandemic, the disease prevention and control (DPC) system takes a lot of responsibilities but also reveals some weaknesses (3). The DPC system suffers from both temporary and permanent workforce shortages (4, 5). The absolute shortage of DPC staff, an ongoing problem within the system, has been discussed in many studies, so it will not be repeated here (6–8). Our study focuses on the relative shortage of DPC staff, which is caused by the surging workload of the fight against the pandemic and, on the other hand, is reflected in the demand for workers of specific discipline(s) and the resulting work stress. How to implement an efficient DPC human resource reserve, keeping the workforce knowledgeable and capable so that their job responsibilities can be effectively complemented and transformed at ordinary times and in case of a serious epidemic is the problem that our study wants to explore.

Beijing City, (with more than 21 million permanent residents, has faced several bouts of the COVID-19 epidemic with timely and effective measures. These outbreaks derived from diverse sources, from the imported cases from Wuhan in January 2020, the imported cases from abroad in late February, to the cluster of cases in Xinfadi Market (a major agricultural products and seafood wholesale market in Beijing) on June 11 (9). During the implementation of the Dynamic zero-COVID approach of China, the municipal (648 staff) and district levels (754 staff) DPC mechanism was well-performed, and the vast majority of tasks were completed by staff in the local DPC system, except the affairs at the level of expert consultation and decision-making, for which a few of external support was resorted (10). Therefore, the practices of Beijing in allocation and cross-complementation of workforce by disciplines and duties are valuable and referential. In our study, the professional staff of different disciplines in the Beijing DPC system were surveyed and analyzed for duties, cross-complementation and work stress during the epidemic, in order to provide a scientific basis for personnel allocation and human resource reserve in respect of infectious disease prevention and control in the DPC system.

## Subjects and method

### Subjects

The cross-sectional survey was made in Apri-May 2021 by way of typical + cluster sampling on professional staff in the Beijing center for disease prevention and control (CDC) and five district-level CDCs of Beijing, who participated in COVID-19 epidemic prevention, control, and disposal at different stages. An electronic questionnaire was adopted in the survey, and 1,127 respondents were collected, suggesting a response rate of 77.5%. Ultimately, 1,086 respondents (96.4%) were considered valid after evaluating and screening based on the object, organization, survey cycle time, and respondence time (≥1 min), as well as the options for related questions. Among the 1,086 respondents, 1,032 staff who directly participated in the COVID-19 prevention and control were selected as the object of our study. With women in the majority (68.99%), the selected staff are mainly aged 31–40 (43.28%) and 41–50 (27.53%), with intermediate titles (45.93%) and associate senior titles (23.35%).

### Survey scope and method

The questionnaire was designed by the Study Group and involves three dimensions, i.e., General Information, Working Intensity, Satisfaction, and Need for Key Capacity Building (Supplementary material). This paper focuses on the former two dimensions: General Information (including the respondent's discipline and duties at ordinary times and during the COVID-19), and Working Intensity and Satisfaction (the working load/intensity in the respondent's perception). The workload mainly comes from the subjective feelings of respondents, which is divided into four levels: low load, average load, full load, and overload.

### Quality control

A field survey was conducted with the support of the Project Team in Beijing CDC, and on the relevant personnel of organizations sampled with the coordination of their administrative staff. The respondents completed the questionnaire on www.wjx.cn and submitted it directly online. Two members of the Study Group independently made an examination and logic check on the data information exported and submitted the inconsistent information to the Project Team for review and verification.

### Statistical analysis

Microsoft Excel 2010 was applied for building a database, and SPSS 22.0 was applied for statistical analysis. Our study is based on categorical data, which are expressed in absolute quantities and proportions. Assuming that the overload of work stress is brought by incremental duties and cross-discipline tasks, a binary logistic regression model is constructed. The dependent variable is overload work stress, which is the subjective feelings of the respondents during the COVID-19 epidemic prevention and control period. Independent variables are duty quantity (the number of duties undertaken by the respondent) and cross-discipline task (tasks undertaken by respondents belonging to N-P, but excluding the virus detection work done by the staff of health inspection discipline, and other COVID-related work done by the staff of non-health-related disciplines). Control variables include gender, age group, professional title, routine work stress, and category of discipline.

## Results

### Distribution of disciplines and duties

**Figure 1:**
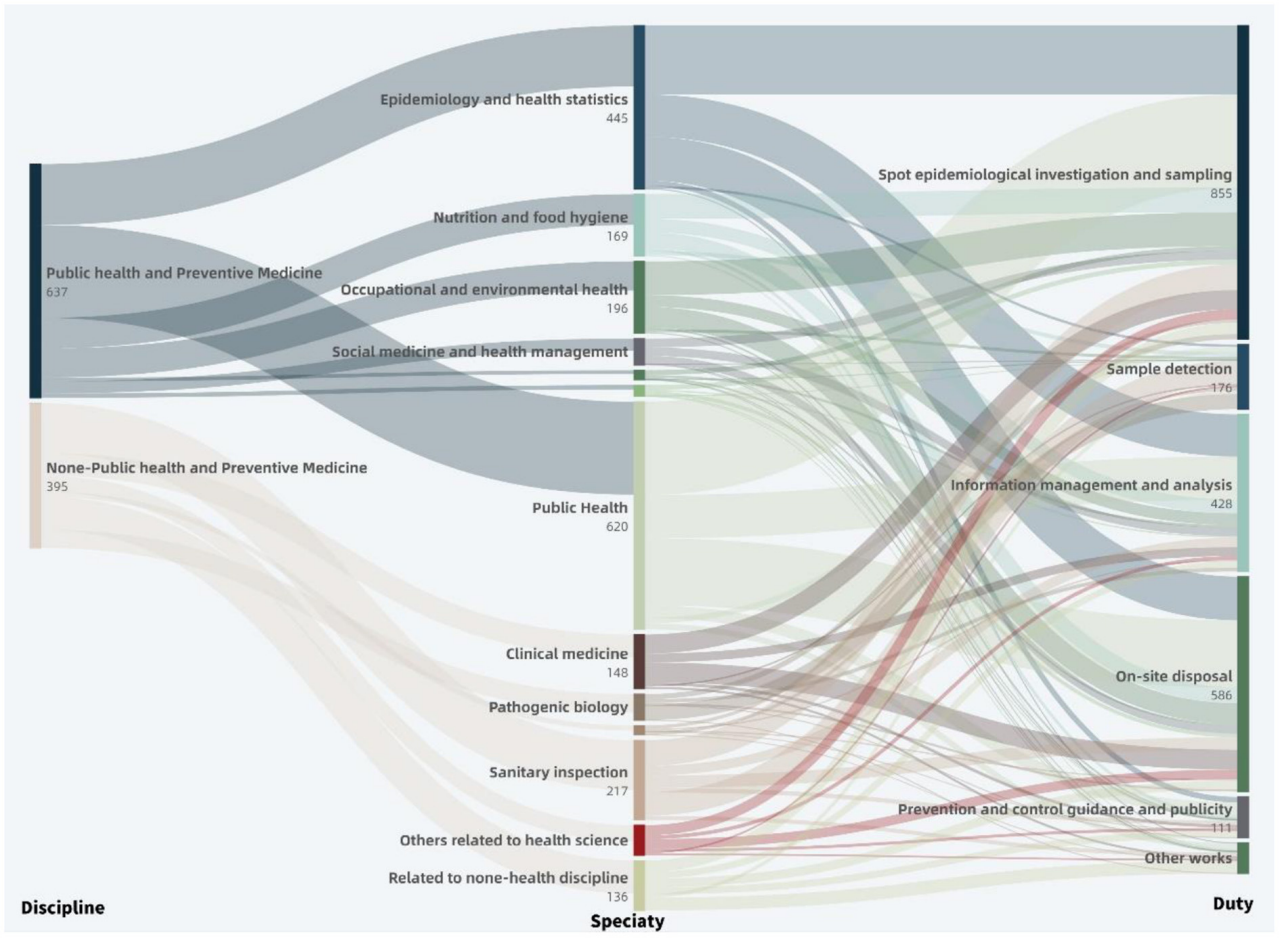
Distribution of discipline and duty during COVID-19 pandemic of frontline staff within Beijing Disease Prevention and Control System. *Discipline*→*Specialty* shows the specific majors distribution of public health and non-public health disciplines (one person can have multiple special majors); *Specialty*→*Duty* shows the distribution of these specific majors act on different epidemic prevention and control duties.

The 1,032 front-line staff include 637 P staff (61.72%) and 395 N-P staff (38.28%) (Figure 1-*Discipline*). The P group is divided into subgroups by 7 specialties, i.e., Epidemiology and Health Statistics (26.06%), Nutrition and Food Hygiene (13.19%), Occupational and Environmental Health (12.40%), Social Medicine and Health Administration (5.18%), Hygiene Toxicology (1.57%), Preventive Medicine (1.88%), and Public Health (39.72%). The N-P group is divided into subgroups by 6 specialties, i.e., Clinical Medicine (15.44%), Pathogenic Biology (12.66%), Immunology (3.54%), Sanitary Inspection (34.43%), Others related to Health Science (11.14%), and Others related to Non-Health Discipline (22.78%) (Figure 1-*Specialty*).

**Figure 2:**
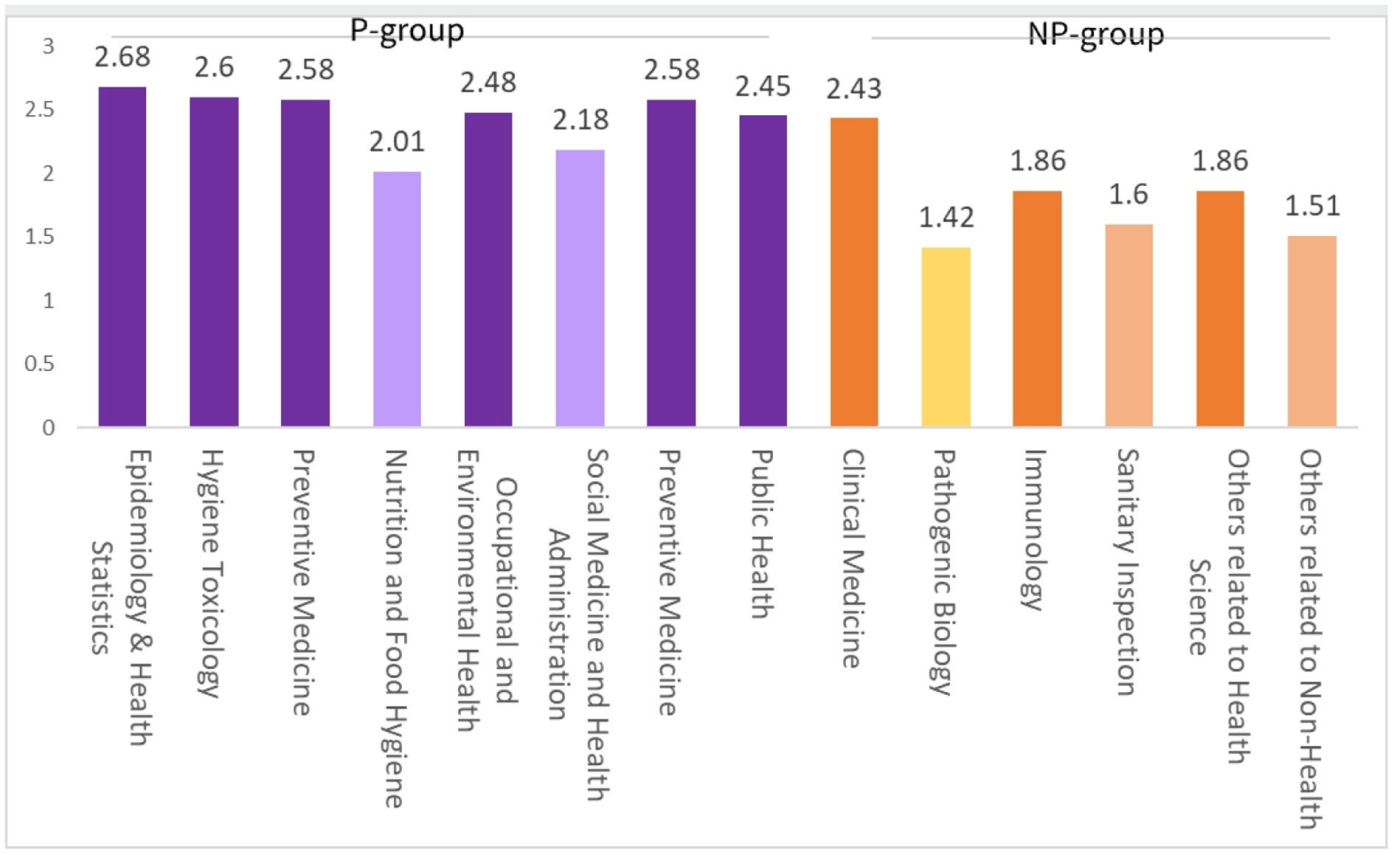
Distribution of epidemic prevention and control duties per-person in different disciplines during COVID-19 pandemic within Beijing Disease Prevention and Control System.

During the COVID-19 epidemic, the 1,032 front-line staff assumed a total of 2,239 duties, that is, 2.17 per person (PP) (Figure 1-*Duty*). The P group reveals an average of 2.45 PP, with the highest recorded by the Epidemiology & Health Statistics subgroup (2.68 PP), followed by the Hygiene Toxicology subgroup (2.60 PP) and Preventive Medicine subgroup (2.58 PP), and the lowest seen in the Nutrition and Food Hygiene subgroup (2.01 PP). The N-P group reveals an average of 1.72 PP, with the highest recorded by the Clinical Medicine subgroup (2.43 PP), followed by the Others related to Health Science subgroup (1.86 PP) and Immunology subgroup (1.86 PP), and the lowest seen in the Pathogenic Biology subgroup (1.42 PP) (Figure 2).

By duties, the Spot Epidemiological Investigation and Sampling takes the highest proportion (38.19%), followed by On-site Disposal (26.17%), Information Management and Analysis (19.12%), Sample Detection (7.86%), Prevention and Control Guidance and Publicity (4.96%), and Other Works (3.71%).

The cross-over analysis of duties and disciplines indicates that in view of Spot Epidemiological Investigation and Sampling, Information Management and Analysis, On-site Disposal and Prevention, Control Guidance and Publicity, the P group accounts for 76.14, 78.50, 74.74, and 57.66%, respectively, higher than the N-P group which accounts for 23.86, 21.50, 25.26, and 42.34%, respectively. In view of Sample Detection and Other Works, the P group accounts for 25.00 and 31.33%, respectively, lower than the N-P group which accounts for 75.00 and 68.67%, respectively. By specialties, the Public Health and Epidemiology and Health Statistics subgroups rank the tops in light of Spot Epidemiological Investigation and Sampling, Information Management, and Analysis, and On-site Disposal duties, with a total proportion higher than 50%; the Sanitary Inspection and Pathogenic Biology subgroups are superior in Sample Detection, with a total proportion up to 59.66%; as to Prevention and Control Guidance and Publicity, the Public Health and Epidemiology and Health Statistics subgroups demonstrate high proportions, being 30.63 and 13.51%, respectively, while the Others related to Non-Health Discipline subgroup (17.12%) takes a non-negligible role; in Other Works, the Others related to Non-Health Discipline subgroup plays a dominant role (42.17%), and the Public Health subgroup also makes a certain contribution (19.28%) (Figure 1-*Duty*).

### Distribution of work stress

**Figure 3:**
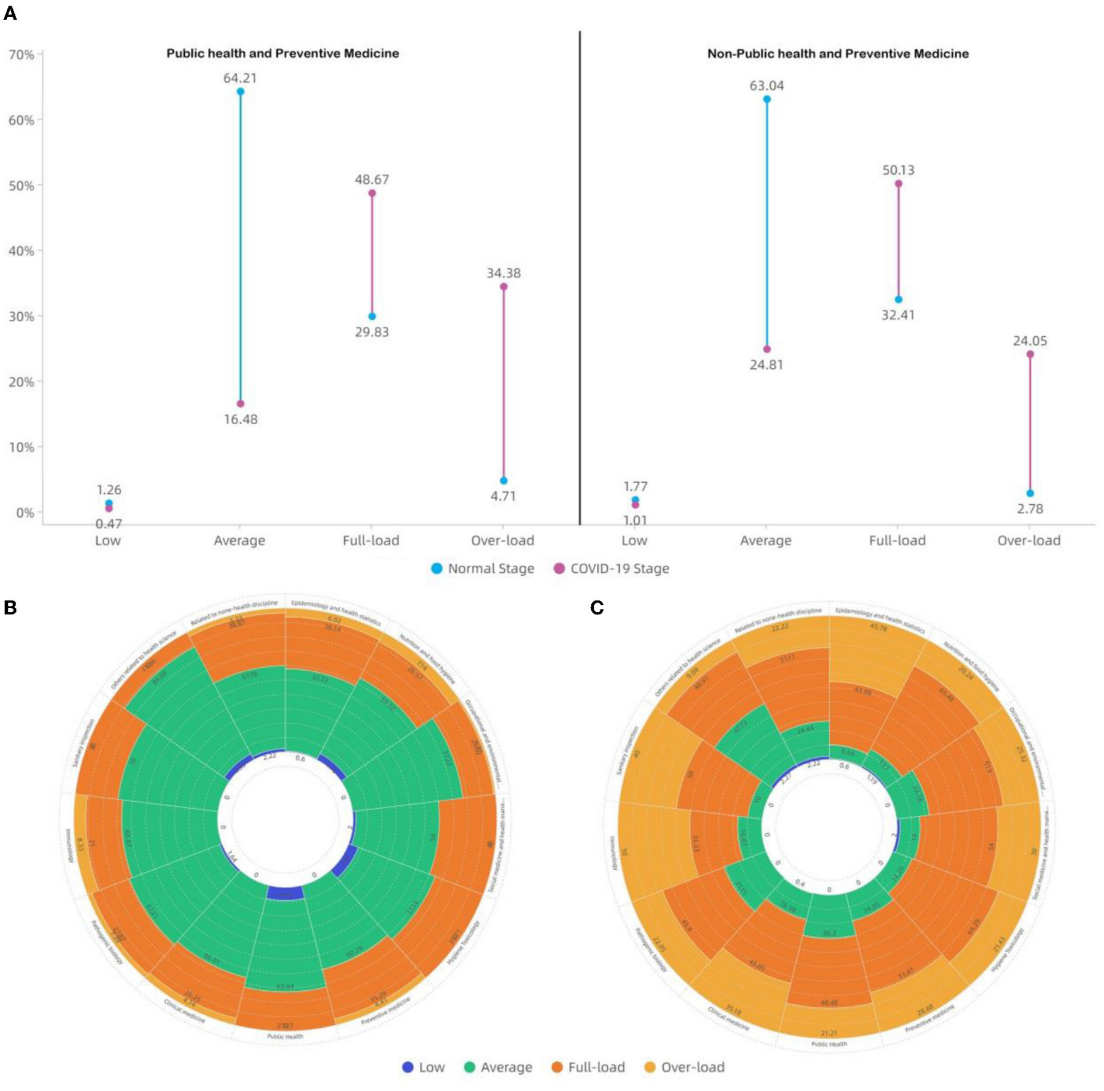
Distribution of work stress in different disciplines before and during the COVID-19 pandemic within the Beijing Disease Prevention and Control System. **(A)** Shows the change in different work stress levels between Normal stage (pre-COVID-19 pandemic) and COVID-19 stage in the P group and N-P group. **(B)** Shows the works stress level in different disciplines during the normal stage. **(C)** Shows the works stress level in different disciplines during the COVID-19 stage.

The analysis reveals that the P group and N-P group have similar proportions in view of full load work stress during the epidemic, being 48.67 and 50.13%, respectively, and the P group shows a proportion of 34.38% in view of overload work stress, apparently higher than the N-P group (24.05%) (Figure 3A). Moreover, both groups indicate their work stresses are higher than the pre-COVID-19 period levels (Figures 3B,C). For the overload of work stress particularly during the COVID-19 epidemic, in the P group, the Epidemiology and Health Statistics subgroup shows the highest proportion (45.78%), while other subgroups fall in the range of 20–30%; in the N-P group, the Immunology subgroup registers the highest proportion (50.00%), followed by the Sanitary Inspection subgroup (40.00%) and the Clinical Medicine subgroup (35.18%).

**Figure 4:**
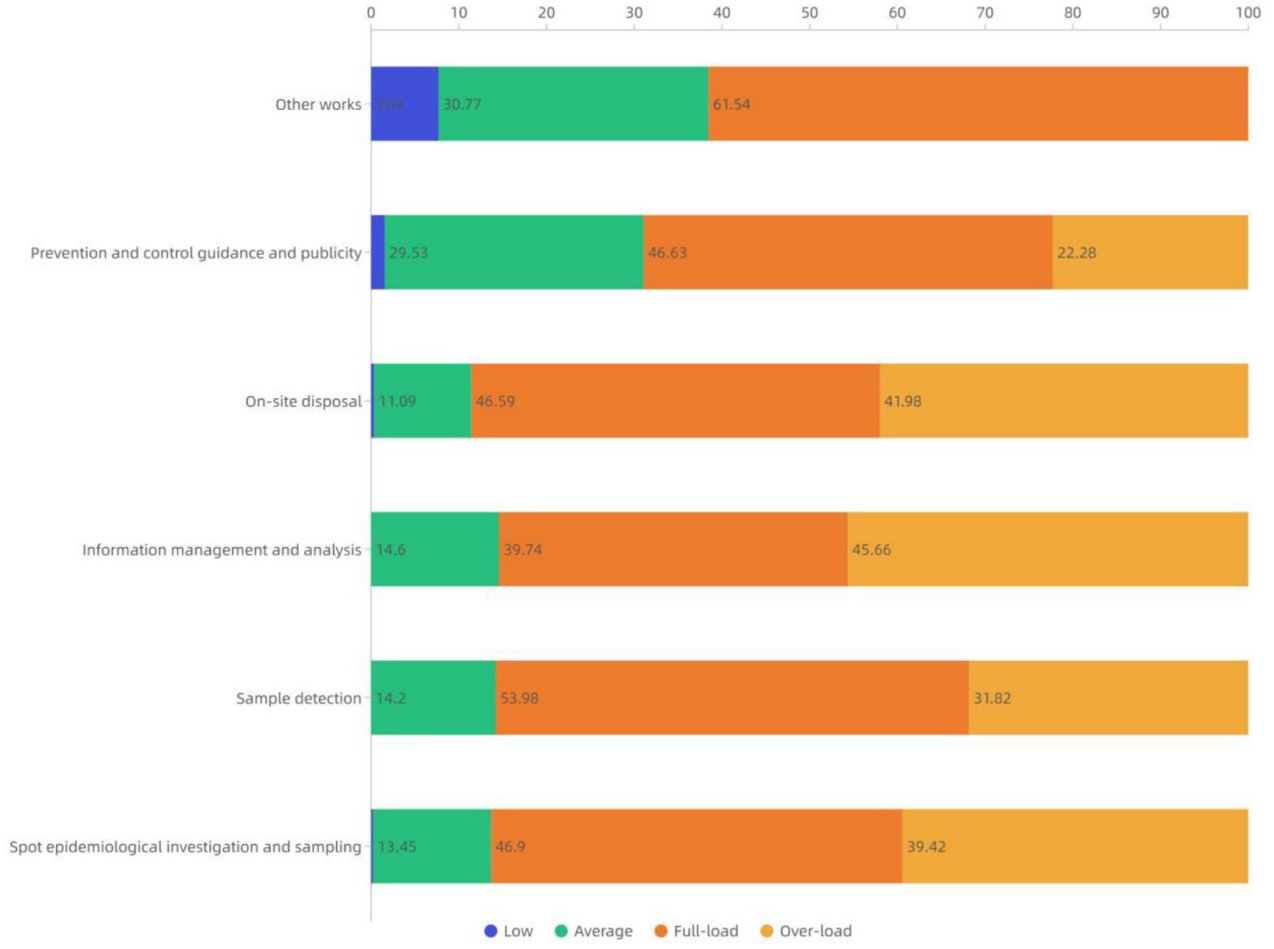
Distribution of work stress in different epidemic prevention and control duties during COVID-19 pandemic within Beijing Disease Prevention and Control System.

All front-line staff on various duties declared high work stress during the COVID-19, indicating the total proportion for full load and overload work stress of 60% or higher. For each of the Information Management and Analysis, On-site Disposal, Spot Epidemiological Investigation and Sampling, and Sample Detection duties, the total proportion for full load and overload work stress exceeds 85%. Specifically, Information Management and Analysis was the highest for overload-induced work stress (45.66%), while the Sample Detection is the lowest for overload work stress but the highest for full load work stress (53.98%). Compared with the above-mentioned four duties, the Prevention and Control Guidance and Publicity shows a similar proportion for full load work stress (46.63%) but a much lower proportion for overload-induced work stress (22.28%), and demonstrates a higher proportion for ordinary load work stress (29.53%). The Other Works represents work stress mainly from full load (61.54%) and ordinary load (30.77%) (Figure 4).

### Work stress impact factors

The duty quantity and cross-discipline task are statistically positively correlated with the probability of overload work stress (*P* < 0.05 and β > 0). The assumption of one more duty causes the probability of overload to increase by 58% (β = 0.58), or by 41% (β = 0.41) in the case of a cross-discipline task. Also, some other factors are significantly related to workload. For example, the higher the age, the higher the probability of overload was declared. The group with primary professional titles reports a higher probability of overload than the group with other levels of titles, and the level of routine work stress has a negative impact on work stress during COVID-19; that is, the staff under higher routine work stress is more tolerant of the additional work stress during the COVID-19 epidemic stage (Table 1).

**Table 1:** Analysis on influencing factors of overload occupational stress during COVID-19 within Beijing disease prevention and control system.

**Factors**	**B**	**S.E**,	**Wals**	**df**	***P*-value**
Sex = Female (Male = 0)	−0.02	0.17	0.02	1.00	0.89
**Age groups (under 30** **=** **0)**			12.41	3.00	0.01
31–40	0.89	0.44	4.10	1.00	0.04
41–50	1.08	0.34	9.85	1.00	<0.01
51–60	1.10	0.32	11.57	1.00	<0.01
**Professional post (No post** **=** **0)**			10.94	4.00	0.03
Primary	1.14	0.50	5.13	1.00	0.02
Intermediate	0.25	0.44	0.31	1.00	0.57
Vice senior	0.08	0.40	0.04	1.00	0.84
Senior	−0.07	0.36	0.04	1.00	0.84
**Daily working pressure (Low** **=** **0)**			88.52	3.00	<0.01
Average	−5.47	1.29	18.06	1.00	<0.01
Full-Load	−4.50	0.75	35.94	1.00	<0.01
Overload	−3.22	0.75	18.36	1.00	<0.01
Discipline = P (NP = 0)	0.29	0.27	1.14	1.00	0.28
Number of prevention jobs	0.41	0.05	61.61	1.00	<0.01
Cross discipline	0.58	0.29	3.95	1.00	0.05
Constant	0.42	0.94	0.20	1.00	0.65

## Discussion

During the COVID-19 epidemic, the workload allocation was apparently dependent on disciplines/specialties. The P group did more work and played a more dominant role in core duties. Public Health, Epidemiology, Health Statistics, and Sanitary Inspection are major essential specialties for a DPC system. In our study, more than 50% of the frontline professional staff are covered by these specialties. Moreover, these three specialties have always been core components in a DPC system, in respect of either talent introduction or personnel training, and made great contributions to the fight against the epidemic. The P group is also found to be efficient support on duties that require high levels of expertise and skills. For such duties as Spot Epidemiological Investigation and Sampling and On-site Disposal, the Nutrition and Food Hygiene subgroup can act as a cross-complement; for Information Management and Analysis, the Social Medicine and Health Management and Occupational and Environmental Health subgroups can be a good standby.

The N-P group is also indispensable in the fight against the epidemic. The surging workload in epidemic prevention, control, and medical treatment brought a great impact on the allocation of health human resources. In this aspect, many researchers have discussed the feasibility of completing certain tasks by external or non-professional staff and analyzed relevant practices, indicating that non-professional staff can be trained properly to fit certain epidemic control duties (11, 12). This conclusion agrees with our study result. It is found that the N-P group took charge of around 30% of duties in the fight against COVID-19, excluding those related to inspection, so the N-P group is an important workforce in the event. Its effective and reliable role can be evidenced by the good COVID-19 epidemic control performance in Beijing.

During the epidemic, the professional staff in the DPC system universally worked under stress from full load and overload. Generally, work stress refers to the stress caused by work or factors in direct relation to work. Such factors include too much work, job change, time pressure, heavy work duty, role change, too high requirements of job-aided hardware/devices for personal skills, irregular working hours, shifts, and work in poor natural and social environments, etc. Excessive work stress may lead to fatigue, anxiety, depression, reduced ability to work, and even burnout (13–15). The Information Management and Analysis, for example, reveals the highest proportion of overload-induced work stress, depending on its working style and method. Through interview with front-line staff working for data management and analysis, we find that they felt higher work stress mainly due to surging workload, time-based feedback of analysis results, long-term work in shifts, and lack of software and knowledge about statistics and database management and analysis. High work stress wore or burnt them out, and made them passionless or even antagonistic to their work, which is adverse to the sustainability of relevant duties (10). We quantitatively identify the stress sources only from the perspective of duty quantity and cross-discipline task, which, however, have certain differences from outside environment stress sources (e.g., risk of infection, and shortage of protective materials) for medical and healthcare workers (16, 17). Different from the working styles of medical and healthcare workers, the front-line DPC staff often need to take charge of several duties and are challenged by a mismatch of discipline, compelling them to study and accommodate as quickly as they can.

Analysis of factors for the work stress of professional staff during the epidemic can provide a reference for personnel allocation and human resource reserve in the DPC system. The work scope and responsibilities of the DPC system decide it as a complex multi-discipline personnel structure with the P staff at the core (18–20). As the COVID-19 pandemic out broke, the entire public health system was exposed to a shortage of human resources. Experts suggest sustaining a sufficient and sustainable health workforce in case of an unexpected epidemic by (1) lifting the restrictions on the employment of health professionals through legal means and expanding the services that health professionals can provide; (2) carrying out professional training for backup personnel to enable them to master corresponding skills and knowledge in a short period of time; and/or (3) recruiting qualified talents from medical students and retired professionals (11). As to the cultivation and talent reserve of professional staff, especially DPC staff, some experts have proposed the “T”-shape talent model, namely, a talent should have a solid knowledge and application ability of their discipline and also be knowledgeable about epidemic prevention and control (21), allowing them to transform smoothly between role at ordinary times and role during an epidemic. Our study results indicate that the prevention and control of any epidemic requires at least twice as many professional staff as usual. Mitigation of overload-induced work stress can be realized by reducing the number of duties a staff takes and the cross-discipline services. Multidisciplinary talents serve as critical support to routine DPC efforts. Merely expanding the P team while neglecting the human resources of other disciplines is inadvisable and impractical. As to personnel allocation and human resource reserve in respect of infectious disease prevention and control, two solutions are optional: less addition of P staff, and more addition of N-P staff. A balance between P and N-P staff that enables the personnel composition to accommodate both routine DPC and unexpected epidemics needs to be further discussed.

The main strength of this study is we use the survey data to comprehensively analyze the objective workload and subjective stress of the front-line staff of the DPC during the COVID-19 epidemic. Despite the findings above, our study is deficient in four aspects. First, the study object is just set in the municipal and district-level DPC systems of Beijing. Horizontally, it is not a perfect regional representative, with its experience not sufficient for extending to the DPC sectors of various public health systems. Vertically, the analysis of professional staff in the DPC systems at the state level and the lower-than-municipal/district levels remains a gap;in actuality the work scope and styles of such professional staff are very different from the staff in provincial/municipal PDC system. Second, the cross-discipline task is defined only roughly. It is noteworthy that some specialties in the P discipline cannot effectively support the tasks in actual epidemic prevention and control. Our study failed to differentiate the specialties due to the availability of data. Third, our study only covers limited factors for work stress but does not incorporate the recognized factors such as absolute workload, time pressure, excessive heavy work duties, and irregular working hours, due to the limitations of the survey method and scope. A specific survey on work stress will be expected as more systematic and thorough data are collected. Fourth, the discipline classification is relatively chaotic in this study. The reason is the discipline classification was mainly based on the degree-related majors of the respondents. If they graduated from the school of public health and obtained a bachelor's degree, they will write their major in preventive medicine, but if they obtain a master's or doctoral degree, they will have more detailed and specific majors, such as occupational health, nutrition, etc. But there are also professional master's degrees awarded in the major of public health. We hope to establish a more scientific and practical discipline classification pattern in future research.

In order to combat such pandemic situations, the training and teaching of the DPC workforce should be modified at a more fundamental level. In particular, undergraduate education and Vocational Continuing Education should focus on the knowledge and skills of epidemic prevention and control, so as to ensure that personnel have sufficient knowledge reserves to deal with the epidemic.

## Conclusion

The front-line staff in the DPC system involved in COVID-19 prevention and control primarily falls into the category of Public Health and Preventive Medicine discipline. The P group assumes the primary or even overload-induced work stress most duties, and the N-P group serves as an important cross-complement. The study results indicate that the prevention and control of any same-scale epidemic will double the amount of work of the Public Health and Preventive Medicine discipline staff. As to personnel workforce recruitment, allocation, and human resource reserve in respect of infectious disease prevention and the DPC system, two solutions are optional: less addition of P staff, and or more addition of N-P staff. A balance between P and N-P staff that enables the personnel composition to accommodate both routine DPC and unexpected epidemics needs to be further discussed.

## Data availability statement

The original contributions presented in the study are included in the article/Supplementary materials, further inquiries can be directed to the corresponding author/s.

## Ethics statement

Ethics approval and written informed consent were not required for this study in accordance with national guidelines and local legislation.

## Author contributions

AM is responsible for research design, data analysis, and thesis writing. YY, SJ, and YM participated in data analysis and thesis writing. QX participated in the translation of the questionnaire and the revision of some figures. WQ and SJ participated in project design and coordination of research activities. All authors contributed to the article and approved the submitted version.

## Funding

This project was funded by Capital Health Development Scientific Research project a (2021-1g-4251) and Peking Union Medical College Education Foundation Project.

## Conflict of interest

The authors declare that the research was conducted in the absence of any commercial or financial relationships that could be construed as a potential conflict of interest.

## Publisher's note

All claims expressed in this article are solely those of the authors and do not necessarily represent those of their affiliated organizations, or those of the publisher, the editors and the reviewers. Any product that may be evaluated in this article, or claim that may be made by its manufacturer, is not guaranteed or endorsed by the publisher.
