# Supplementary material for: Understanding the condition of disease prevention and control workforce by disciplines, duties, and work stress during the COVID-19 pandemic: A case from Beijing disease prevention and control system
Source: Front Public Health. 2022 Aug 18;10:861712. doi: 10.3389/fpubh.2022.861712 (PMC9433976; doi:10.3389/fpubh.2022.861712)
Supplement: Supplementary file 1 [file Data_Sheet_1.docx]

**Questionnaire on capacity building of professional technicians in Centers for Disease Prevention and Control**

**PART Ⅰ: Basic information**

**Q1.Your working institution**: **__________________**

**Q2.Your Gender: _________**

①Male ②Female

**Q3.Your Age (years old): ________**

①<30 ②31-40 ③41-50 ④51-60 ⑤>60

**Q4.Your highest degree: _________**

①Below bachelor degree ②Bachelor degree ③Master degree

④Doctoral degree or above

**Q5.Your subject category: ___________**

1. Epidemiology and Health Statistics
2. Nutrition and Food Hygiene
3. Occupational Hygiene and Environmental Hygiene
4. Pathogen Biology
5. Immunology
6. Sanitary Analysis
7. Social Medicine and Health Service Management
8. Public Health
9. Clinical Medicine
10. Other______________

**Q6.Your job title: __________**

①Senior title ②Vice-senior title ③Middle title ④Primary title

⑤None ⑥Other______________

**Q7.Your responsibilities at work are________ (multiple choices are allowed)**

①Prevention and control of infectious disease

②Prevention and control of non-communicable diseases

③Prevention and control of endemic

④Prevention and control of parasitic diseases

⑤Disinfection and vector prevention and control

⑥Immunization program

⑦Food safety

⑧Monitoring and evaluation of occupational, radiological and environmental disease

⑨School hygiene preventive controls

⑩Emergency management of public health emergencies

⑪Health education and health promotion

⑫Pathogenic microorganisms and biological testing

⑬Physical and chemical testing

⑭Toxicological testing and evaluation

⑮Information and network management

⑯Business management and quality control

⑰Others______________

Q8. During the COVID-19 outbreak, your responsibilities for prevention and control are: ________(multiple choices are allowed)

①Not involved in related work

②Epidemiological investigation

③Sample collection and delivery

④Sample testing

⑤On-site disposal

⑥Information monitoring and statistical analysis

⑦Database management

⑧Epidemic analysis and judgment

⑨Medical observation

⑩Disinfect

⑪ Other

**PART Ⅱ: Work intensity and satisfaction**

**Q9.** **What is your usual workload? _______**

1. Low load ②Average load ③Full load ④Overload

**Q10.What is your workload during the epidemic prevention and control period? _______**

1. Low load ②Average load ③Full load ④Overload

**Q11.Are you satisfied with the work arrangement during the epidemic prevention and control period? _______**

①Very satisfied ②Generally satisfied ③Average

④Unsatisfied ⑤Very unsatisfied

**Q12.** **Are you satisfied with the logistical support during the epidemic prevention and control period? _______**

①Very satisfied ②Generally satisfied ③Average

④Unsatisfied ⑤Very unsatisfied

**Q13.** **Are you satisfied with your usual salary level? _______**

①Very satisfied ②Generally satisfied ③Average

④Unsatisfied ⑤Very unsatisfied

**Q14. Are you satisfied with the temporary work allowance given during the epidemic prevention and control period? _______**

①Very satisfied ②Generally satisfied ③Average ④Unsatisfied

⑤Very unsatisfied ⑥Have not received relevant subsidies, do not understand

**Q15. Are you satisfied with the job title promotion system of your work place? _______**

①Very satisfied ②Generally satisfied ③Average

④Unsatisfied ⑤Very unsatisfied

**Q16. Have you ever thought of leaving your job? _______**

①No ②Yes

**Q17. The reasons for wanting to leave are: _______ ( multiple choices are allowed)**

①Stress from work

②Low salary

③Difficulty in promotion

④The assessment and incentive mechanism is unreasonable

⑤Development prospects are not optimistic

⑥Self-worth cannot be reflected

⑦Poor working environment

⑧Personal family reasons ⑨Other______________

**PART Ⅲ: Core competencies**

**Q18.Please judge whether the following core competencies are required for daily work, and whether you already possess them.**

**(1=not required and not possess, 2=not required but possess, 3=not sure, 4=required and possess, 5=required but not possess)**

| **Core competencies** | **self-judgment**  （Please fill in the corresponding number "1-5"） |
| --- | --- |
| Basic knowledge of epidemic prevention and control |  |
| Basic skills for epidemic prevention and control |  |
| Epidemiological investigation capacity |  |
| Emergency response capability |  |
| Laboratory testing capabilities |  |
| Detection method development capabilities |  |
| Integration and analysis capabilities of large databases |  |
| Complex mathematical model modeling ability |  |
| Big data acquisition and analysis capabilities |  |
| Data mining capabilities |  |
| Ability to select scientific research topics (scientific research topics related to epidemic prevention and control for this position) |  |
| Use cross-domain knowledge and skills to assist epidemic prevention and control capabilities |  |
| Communication and coordination ability |  |
|  |  |
|  |  |

**Q19.In order to further improve the ability to prevent and control infectious diseases, what training programs or scientific research projects do you think are urgently needed in the current work?(** **Names of specific projects to be undertaken can be listed)**
